# The Effect of Missing Item Data on the Relative Predictive Accuracy of Correctional Risk Assessment Tools

**DOI:** 10.1177/10731911231225191

**Published:** 2024-02-07

**Authors:** Bronwen Perley-Robertson, Kelly M. Babchishin, L. Maaike Helmus

**Affiliations:** 1 Carleton University, Ottawa, Ontario, Canada; 2 Simon Fraser University, Burnaby, British Columbia, Canada

**Keywords:** correctional risk assessment, missing data, predictive accuracy, sexual recidivism, domestic violence recidivism

## Abstract

Missing data are pervasive in risk assessment but their impact on predictive accuracy has largely been unexplored. Common techniques for handling missing risk data include summing available items or proration; however, multiple imputation is a more defensible approach that has not been methodically tested against these simpler techniques. We compared the validity of these three missing data techniques across six conditions using STABLE-2007 (*N* = 4,286) and SARA-V2 (*N* = 455) assessments from men on community supervision in Canada. Condition 1 was the observed data (low missingness), and Conditions 2 to 6 were generated missing data conditions, whereby 1% to 50% of items per case were randomly deleted in 10% increments. Relative predictive accuracy was unaffected by missing data, and simpler techniques performed just as well as multiple imputation, but summed totals underestimated absolute risk. The current study therefore provides empirical justification for using proration when data are missing within a sample.

Correctional risk assessment is vital to public safety and the fair treatment of those being evaluated, as it directly informs sentencing and release decisions (Monahan & Skeem, 2014). The validation of risk tools has therefore become a prolific correctional task, but relatively little attention has been paid to missing data. This is surprising given the prevalence of missing data in risk assessment arising from incomplete item information, which can change the composition of risk tools. For instance, recent research used a modified version of the Spousal Assault Risk Assessment (SARA) because three to six items could not be scored from file information (Jung & Buro, 2017; Jung et al., 2022; Olver & Jung, 2017). Studies of other risk tools have similarly excluded items with insufficient information to score (Etzler et al., 2020; Nunes et al., 2002). In prospective research, front-line correctional and mental health staff have omitted items when scoring the SARA (Kropp & Hart, 2000), the STABLE-2007 (Hanson et al., 2015), and the Violence Risk Scale-Sexual Offense Version (VRS-SO; Olver et al., 2014). In short, missing data is a commonplace challenge in research and practice.

Changing the composition of risk tools to accommodate missing data could degrade predictive accuracy if the strongest predictors are omitted. In addition, risk is a probabilistic, continuous dimension that follows a cumulative stochastic model (Hammond & O’Rourke, 2004; Hanson et al., 2013). This means that individual factors are not deterministic in their contribution to risk (Helmus & Babchishin, 2017). As such, risk assessment results based on the complete set of items should lead to more accurate predictions compared to results based on a subset of scale items.

Although early researchers attributed suboptimal predictive accuracy results to missing items, the evidence thus far is lacking. Harris et al. (2003) found better predictive accuracy for four risk tools after deleting incomplete cases, but this method is known to produce distorted parameter estimates when data are missing systematically (Enders, 2010; Little & Rubin, 2002). Paradoxically, Hanson and Morton-Bourgon (2009) reported better predictive accuracy among studies in their meta-analysis with more missing data, but this could be due to biased missing data handling techniques in the primary studies. Other researchers reported no correlation between missing data and the predictive accuracy of risk tools (Smid et al., 2014), but they deleted cases with more than 20% missing data, potentially biasing their results (Enders, 2010; Little & Rubin, 2002).

Evaluating the impact of missing data is further complicated because not many studies address this. For instance, only 28% of studies in one risk tool meta-analysis reported the amount of missing data (Hanson & Morton-Bourgon, 2009). A systematic review also found that researchers were unclear about the methods used to address missing risk assessment data (Tully et al., 2013). This is a major gap in the literature, which the current study addresses by comparing different approaches for calculating risk scores when items are missing. The first two approaches are standard practice in risk assessment, and the third is an advanced alternative that may be more suitable in research contexts. These methods are compared under different missing data conditions using two popular risk tools for sexual and domestic violence recidivism: the STABLE-2007 and the SARA, Version 2 (SARA-V2).

## Missing Data Approaches

### Mechanical Total Scores

A common approach for calculating total scores is to simply sum the available items, particularly when the coding rules do not provide guidance on handling missing information. This approach has been used for mechanical tools, which rely on scale total scores to communicate risk, such as the STABLE-2007 (Helmus et al., 2021). It has also been used for structured professional judgment (SPJ) tools like the SARA, but only for research purposes (Jung & Buro, 2017; Jung et al., 2022). This is because SPJ tools rely on evaluators’ summary risk ratings to communicate risk (e.g., low, moderate, and high), which are based on professional opinion without summing the items (Hanson & Morton-Bourgon, 2009). To avoid the loss of precision that comes with categorizing a continuous construct, researchers commonly calculate total scores when validating SPJ tools.

Calculating total scores by ignoring missing items is convenient but can underestimate risk because a score of zero is assumed for missing items (Downey & King, 1998). This could reduce the association between scale scores and recidivism for those who reoffended and would have scored higher than zero on missing items. For example, if a recidivist’s true risk score is 20/20, but they were missing half the items, their mechanical total would be 10/20. Ignoring missing items by summing without correction also redefines the composition of the scale by the missing data patterns and rates unique to a given sample (Schafer & Graham, 2002 discuss this issue for proration, but the same logic applies here). For instance, suppose that Sample A, Sample B, and Sample C were scored on a 10-item risk scale: everyone in Sample A had complete data; everyone in Sample B was missing Items 1 to 3 and 20% were missing Items 4 to 5; and everyone in Sample C was missing Items 6 to 8 and 60% were missing Items 9 to 10. The composition of the risk scale is now redefined by the missing data patterns and rates unique to each sample. This could reduce predictive accuracy in Samples B or C if missing items are stronger predictors of recidivism than complete items. This would also limit the generalizability of results as sample data are based on different items.

Finally, summing available items assumes data are missing completely at random (MCAR) because ignoring systematic missingness can introduce bias into the results (Baraldi & Enders, 2010; Enders, 2010; Rubin, 1976). The MCAR assumption may not hold in risk assessment because certain factors are more readily available or easier to verify than others. For example, static risk factors (e.g., number of previous convictions, age at first offense) are easier to score retrospectively than dynamic risk factors (e.g., motivation to change, insight, attitudes) because they are commonly recorded in criminal justice databases (e.g., Jung & Buro, 2017; Maltais et al., 2024). It also seems that supervision officers more often report the presence versus absence of dynamic risk factors in their notes, which is especially true for less conventional factors like insight or motivation (Maltais et al., 2024; McLaren et al., 2024). Some missing dynamic items, therefore, could reflect an absence of risk.

Scale items that require mental health assessments might also be missing if such assessments are not part of routine practice. In Canadian provincial corrections, for example, psychological assessments might only be available when there is enough cause for concern to request them (e.g., see Jung & Buro, 2017). Items requiring self-report information might be missing too if the person is not credible and collateral information is not available (e.g., see the scoring rules for the Static-99R and STABLE-2007 item *ever lived with a lover*; Fernandez et al., 2014; Phenix et al., 2016). This can create systematic missingness as well.

### Proration

Another common approach for handling missing item scores is proration (also called person mean imputation). This involves filling in someone’s missing scores with the mean of their available scores (Downey & King, 1998; Schafer & Graham, 2002). Proration is common in correctional risk assessment, with several scales providing prorating instructions, including the Ontario Domestic Assault Risk Assessment (ODARA; Hilton, 2021), Domestic Violence Risk Appraisal Guide (DVRAG; Hilton, 2021), VRS-SO (Wong et al., 2003–2020), and the Violence Risk Appraisal Guide-Revised (VRAG-R; Harris et al., 2015).

For tools that use proration, it is unclear whether scale authors determined the number of allowable item omissions empirically or tested proration against other methods for handling missing data. A review of some development studies, however, suggests prorating policies determined by risk scale developers were not empirically based (Harris et al., 1993; Hilton et al., 2004, 2008; Olver et al., 2007; Rice et al., 2013). This is problematic because, like with mechanical totals, proration redefines the composition of the scale by the missing data patterns and rates unique to the study sample (Schafer & Graham, 2002). Prorated total scores therefore reflect all items for people with complete data and various item subsets for people with incomplete data. This could reduce predictive accuracy if the missing items are stronger predictors of recidivism than available items. It could also limit generalizability, as discussed above for mechanical totals (Schafer & Graham, 2002). Finally, proration assumes data are MCAR (Enders, 2017) but has been shown to produce biased results even when this assumption is met. This is due to the additional assumption that a given respondent will score similarly across scale items (e.g., equal item means; Mazza et al., 2015; Mcdonald et al., 2000). Risk tools, however, often contain distinct domains (e.g., general and sexual criminality; Brouillette-Alarie et al., 2023), which undermines this assumption and may result in over- or under-estimation of risk.

Despite the above limitations, research has found that proration preserved original scale scores when up to 20% of cases were each missing 10% to 40% of items completely at random (correlations between original and prorated scores were .99; Downey & King, 1998). When up to 35% of cases had a maximum of 70% missing data, correlations decreased to .93. Proration in risk assessment may therefore be defensible under certain conditions and it may offer better performance than mechanical totals. This is because mechanical totals assign zeros to missing items, whereas proration takes someone’s contribution to available items and applies it to missing items, aligning their score on missing items with their observed risk level rather than assuming an absence of risk. The robustness of proration found above, however, may be an overestimate when applied to risk assessment. Downey and King’s simulation used norm-referenced scales, where items are presumed to measure a single latent construct. Given that risk assessment scales are criterion-referenced and intended to measure multiple constructs (Brouillette-Alarie et al., 2023; Helmus & Babchishin, 2017), the expected intercorrelations between items should be lower, which should attenuate the effectiveness of proration.

### Multiple Imputation

Multiple imputation (Rubin, 1987, 1996) is a more advanced technique that is widely viewed as one of the best options for addressing missing data (Enders, 2010; Newman, 2014; Schafer & Graham, 2002). Multiple imputation involves generating multiple datasets, each with different estimates of the missing values (Rubin, 1987, 1996). The substantive analyses are run on each imputed dataset and the results are aggregated. These three steps are described as the imputation, analysis, and pooling phases, respectively.

While the analysis and pooling phases are common to all multiple imputation analyses, there are different imputation algorithms for different types of data (e.g., categorical, continuous, longitudinal, multilevel; Enders, 2010). Most impute missing values through a two-step iterative regression procedure (Enders & Baraldi, 2018). In Step 1, observed data are used to construct regression equations that predict the missing values, adding a residual term to predicted scores (otherwise imputed values fall directly on the regression line, artificially reducing variability in scores). The imputed data are then used to estimate new regression parameters in Step 2, which are carried forward to the next iteration. Here, the new regression parameters are used to generate updated imputations (Step 1) and the imputed data are used to estimate yet another set of regression parameters (Step 2). Steps 1 and 2 are repeated, each time updating the relevant values, until the parameter estimate distributions from Step 2 are stable across iterations (i.e., convergence; Enders, 2010).

Imputing data this way provides a good approximation of what they would look like had they been complete (Baraldi & Enders, 2010; Enders, 2010; Newman, 2014; Woods et al., 2023). Aggregating results across multiply imputed datasets also accounts for uncertainty in missing values (Baraldi & Enders, 2010). Unsurprisingly, multiple imputation produces more accurate parameter estimates and standard errors than traditional techniques when data are not MCAR (Baraldi & Enders, 2010; de Goeij et al., 2013; Newman, 2003, 2014; Schafer & Graham, 2002; Woods et al., 2023)—although data are assumed to be at least missing at random (MAR; Rubin, 1976, 1987).

Multiple imputation has only been compared to simpler missing data handling techniques in one risk assessment study to date (Viljoen et al., 2017). These authors examined whether missing data influenced rank-order stability coefficients and correlations between risk change scores and recidivism for two youth risk assessment tools. Multiple imputation was conducted on scale total scores and compared to proration after listwise deletion (i.e., cases missing 10% of items were removed prior to proration). Both techniques performed similarly, but there are several limitations to Viljoen and colleagues’ work. First, they used Little’s MCAR test (Little, 1988) to evaluate the MAR assumption. Despite its widespread use, methodologists have identified numerous issues with this test. One, it is prone to Type 2 errors under certain conditions (Thoemmes & Enders, 2007, as cited in Enders, 2010, p. 21). Two, Little’s is not a conclusive test of MCAR because it relies on mean comparisons, and MAR and Missing Not at Random (MNAR) mechanisms can produce missing data subgroups with equal means (Enders, 2010). Three, Little’s cannot identify specific variables that violate MCAR, meaning it can only be used to test an omnibus hypothesis that rarely holds in practice (Enders, 2010; Muthén et al., 1987; Raghunathan, 2004).

Another methodological limitation of Viljoen and colleagues’ (2017) work includes the imputation of scale total scores instead of items, which reduces statistical power (Gottschall et al., 2012; Mazza et al., 2015). In addition, the authors did not describe their imputation model, making it difficult to evaluate statistical power (Graham et al., 2007), nor did they specify if they included auxiliary variables (discussed in the Method section), which increase power and make inadvertently omitting a cause of missingness less likely (Collins et al., 2001). Viljoen and colleagues’ work therefore does not adequately address the question of whether simple missing data techniques, like listwise deletion and proration, perform similarly to multiple imputation.

## The Current Study

Given the above findings, it seems difficult to justify the use of mechanical totals or proration over multiple imputation when dealing with missing data in risk assessment. Multiple imputation has less stringent assumptions and estimates total scores that should be more representative of people’s true risk level and, consequently, better at predicting recidivism. In the current study, we sought to investigate (a) whether missing data degrade the relative predictive accuracy of two common risk tools for sexual and domestic violence recidivism (STABLE-2007 and SARA-V2), and (b) whether relative predictive accuracy is affected by the missing data handling technique (mechanical totals, proration, and multiple imputation). Six missing data conditions were used to explore these questions. The first was the observed data, which were either virtually complete (STABLE-2007) or had low levels of missingness (SARA-V2). The rest were generated missing data conditions, whereby 1% to 50% of MCAR data were inserted into the observed data in 10% increments. The predictive accuracy of the STABLE-2007 and SARA-V2 was then compared within and across conditions.

We expected mechanical and prorated scores to decrease in predictive accuracy as missing data increased, while predictive accuracy was expected to remain stable with multiply imputed scores. At low levels of missingness (Conditions 1 and 2), the three techniques were expected to perform similarly (Parent, 2013; Schafer, 1999). At moderate to high levels of missingness (Conditions 3 to 6), multiple imputation was expected to perform meaningfully better than the other techniques, while proration was only expected to perform marginally better than mechanical totals.

## Method

### Sample

The dataset used for this study was originally examined in Helmus et al. (2021). Their sample included all men on community supervision in British Columbia, Canada, who received either a Static-99R or STABLE-2007 assessment between January 1, 2005 and June 4, 2013 (*N* = 4,433). We restricted our sample to those with STABLE-2007 assessments (*N* = 4,286) because the Static-99R cannot be scored with missing items (Phenix et al., 2016). We also examined a subsample of men scored on the SARA-V2 (*N* = 455), representing individuals with both sexual and domestic violence offending histories. These two groups are henceforth referred to as the STABLE-2007 and SARA-V2 samples, respectively.

In the STABLE-2007 sample, the mean age at the start of the follow-up was 40.8 years (*SD* = 13.71, range = 18–100). Most men were White (61.1%) or Indigenous (which includes First Nations, Métis, and Inuit; 21.5%), with the rest identified as East Indian (3.6%), Asian (2.7%), Black (1.4%), Hispanic (1.4%), or other (5.2%). Race/ethnicity was missing for 3.2% of the sample. The dataset does not specify how race/ethnicity was defined; likely, it would have often been based on self-report, but not necessarily always. Most of the sample had less than a high school education (43.4%), about one-third completed high school (31.6%), some completed vocational school (8.3%) or university (9.1%), and 7.6% were missing education data.

In the SARA-V2 sample, the mean age at the start of the follow-up was 38.7 years (*SD* = 11.04, range = 19–73). Most men were White (50.1%) or Indigenous (which includes First Nations, Métis, and Inuit; 34.1%), with the rest identified as East Indian (4.4%), Asian (2.4%), Black (2.0%), Hispanic (0.7%), or other (4.4%). Race/ethnicity was missing for 2.0% of the sample. Most of the sample had less than a high school education (50.8%), about one-third completed high school (34.9%), some completed vocational school (8.1%) or university (3.6%), and 2.6% were missing education data. Ethics approval for the secondary use of this dataset was obtained from Carleton University (Clearance #118859) and Simon Fraser University (Clearance #20200133).

### Measures

#### STABLE-2007

The STABLE-2007 (Fernandez et al., 2014; Hanson et al., 2007) is a 13-item mechanical instrument designed to predict sexual recidivism in men convicted of a sexually motivated offense. It comprises stable dynamic risk factors relevant to the treatment and supervision of individuals with sexual offending histories. Each item is rated on a 3-point scale as *no concern*, *some concern*, or *considerable concern*. The coding manual indicates that Item 3 (Emotional Identification with Children) should be left missing if the individual has not committed a sexual offense against a victim less than 14 years old (Fernandez et al., 2014). Otherwise, the user manual does not specify whether items can be omitted if there is insufficient information to score them, but in practice, raters do tend to omit items at their discretion (e.g., Hanson et al., 2015). Total scores (ranging from 0 to 26) are calculated by summing available items and are used to classify individuals into one of three dynamic needs levels (low, moderate, and high). These results can then be combined with a static actuarial instrument (e.g., Static-99R) to classify individuals into one of five overall risk levels and to provide estimated recidivism rates (Brankley et al., 2017). The current study used baseline STABLE-2007 assessments that were completed by community supervision officers. Meta-analytic research shows that the STABLE-2007 discriminates sexual recidivists from nonrecidivists with moderate accuracy (area under the curve [AUC] = .67; Brankley et al., 2021). A little over one-third (39.1%) of the sample was scored on the STABLE-2000, which has three items that differ slightly from the STABLE-2007, three additional items, and a different method for calculating the total score (see Hanson et al., 2007). Previous research has approximated STABLE-2007 scores from STABLE-2000 assessments by removing the three deleted items, approximating the altered items using the 2000 version, and calculating total scores following the 2007 version (e.g., Etzler et al., 2020; Helmus et al., 2021). We used this method in the current study as well.

#### SARA-V2

The SARA-V2 (Kropp & Hart, 2000) is a 20-item SPJ instrument designed to predict intimate partner violence (IPV) recidivism in people who have committed an IPV offense. Items are rated on a 3-point scale (*no/absent*, *possibly/partially present*, and *yes/present*) and measure criminal history, psychosocial adjustment, spousal assault history, and the index IPV offense. These ratings are used in a nonmechanical way to aid clinical judgment in classifying individuals into one of three risk categories (low, moderate, high). Items may be omitted if there is insufficient information to score them; this does not preclude evaluators from reaching a final risk judgment. The current study used baseline SARA-V2 assessments that were completed by community supervision officers. Mechanical SARA-V2 total scores have produced small to large effect sizes in discriminating IPV recidivists from nonrecidivists (Hilton et al., 2004; Jung & Buro, 2017; Kropp & Hart, 2000; Pham et al., 2023).

#### Recidivism

In both samples, new charges or convictions in British Columbia were recorded up until June 4, 2013. The relevant recidivism outcome was any new sexual offense (contact or noncontact) for the STABLE-2007, and any new domestic violence offense for the SARA-V2. The follow-up periods started at conviction date for those with community sentences or release date for those with custodial sentences (if release date was unknown, it was estimated at two-thirds of the sentence). Time to each outcome (or study end date for nonrecidivists) was measured in years. The recidivism date was counted as the offense date, but if this information was not available (<5% of recidivism incidents), it was counted as the earliest known charge date.

The average length of follow-up was 4.5 years (*SD* = 2.51) for the STABLE-2007 sample and 4.3 years (*SD* = 2.38) for the SARA-V2 sample. During this time, 4.7% (*n* = 200) of the STABLE-2007 sample was charged with a new sexual offense, and 30.5% (*n* = 139) of the SARA-V2 sample was charged with a new domestic violence offense. The average time to sexual recidivism was 1.9 years (*SD* = 1.70), and the average time to domestic violence recidivism was 1.2 years (*SD* = 1.38). For more information on the coding of recidivism data and other procedural details, see Helmus et al. (2021).

### Analytic Plan

#### Missing Data Handling Techniques

Mechanical total scores were calculated by summing available items. Prorated total scores were calculated by averaging available items and multiplying this by the total number of scale items (Enders, 2010). Multiple imputation was conducted at the item level using chained equations (i.e., fully conditional specification or sequential regression imputation; Raghunathan et al., 2001; van Buuren, 2007; van Buuren et al., 2006; van Buuren & Groothuis-Oudshoorn, 2011). Multiply imputed total scores were then calculated by summing scale items within each imputed dataset. Chained equations is a common technique for imputing categorical variables that has performed well in numerous simulation studies (Giorgi et al., 2008; Kropko et al., 2014; Moons et al., 2006; Raghunathan et al., 2001; van Buuren et al., 2006). In the current study, chained equations was conducted in R using the *multivariate imputation by chained equations* (MICE) package (van Buuren & Groothuis-Oudshoorn, 2011). Following recommendations by methodologists, we used a minimum of 20 imputations to meet power requirements (Graham et al., 2007) or matched the number of imputations to the percentage of cases with incomplete data when greater than 20% (Bodner, 2008; White et al., 2011). See the Multiple Imputation section of the Online Supplement for more information on this analytic technique, as well as the diagnostic checks performed.

Recall that multiple imputation assumes data are MAR, whereas mechanical totals and proration assume data are MCAR. While there are no remedies if the MCAR assumption of the latter two methods is violated, the MAR assumption can be satisfied in multiple imputation by incorporating missing data correlates into the imputation phase (Baraldi & Enders, 2010; Collins et al., 2001; Enders, 2010). Methodologists also recommend including correlates of the incomplete variables themselves in the imputation phase to help recover some of the lost information. Together, correlates of missingness and correlates of the incomplete variables are referred to as auxiliary variables. Imputation strategies that make minimal use of auxiliary variables are called restrictive, whereas those that make liberal use of auxiliary variables are called inclusive (Collins et al., 2001). Simulation research has demonstrated the benefits of inclusive imputation strategies, including the reduced chance of inadvertently omitting a cause of missingness, reduced bias, and increased power (Collins et al., 2001).

To examine the utility of an inclusive imputation strategy, we tested two models for each scale. The first was a restrictive model that included scale items only (STABLE-2007) or scale items and missing data correlates to satisfy the MAR assumption (SARA-V2).^1^ The second was an inclusive model with added correlates of the incomplete scale items themselves (e.g., other risk scale items, demographics, criminal history). If multiple imputation produces meaningfully better results than the other missing data handling techniques and the restrictive versus inclusive models are similar, future researchers might favor a restrictive model to save time. See the Auxiliary Variables section of the Online Supplement for the auxiliary variables included in our models and how we selected them.

Note that neither model included recidivism as an auxiliary variable. While methodologists advocate for using the outcome variable to predict missing values—saying that any possible inflation of parameter estimates is offset by the variability added to imputed values in the imputation phase (Allison, 2002)—this imputation approach is inappropriate for prognostic assessments. The argument for using outcome variables to impute missing values stems from research on diagnostic assessments. For instance, Landerman et al. (1997) found that excluding the outcome as a predictor of missing values led to spuriously underestimated results when predicting depressive symptoms experienced in the last week. Moons et al. (2006) examined the utility of imputing missing values using the outcome variable in a simulation study on diagnosing pulmonary embolism. They too found that excluding the outcome as a predictor of missing values led to an underestimation of results.

It is therefore not surprising that methodologists recommend following this imputation approach; however, an important distinction must be made between the tasks of prognosis versus diagnosis. Risk tools are prognostic in nature, meaning they are designed to estimate the likelihood that something will occur in the future (Moons et al., 2009). Prognosis is a much more difficult task than diagnosis (identifying something that already exists), which is why acceptable levels of predictive accuracy are much higher in medicine than in risk assessment (Helmus & Babchishin, 2017).

There are many factors that contribute to risk, some of which are known and measured by risk tools, while others are unknown, including idiosyncratic features of the person or their environment (Helmus & Babchishin, 2017). Using recidivism to impute risk scores may inadvertently bias results because information that was not available at the time of assessment is used to inform assessments. This is an impossible scenario and one that researchers typically avoid when coding risk tools retrospectively (i.e., researchers will code risk tools blind to recidivism status to avoid subconsciously rating recidivists as higher risk than nonrecidivists). Using recidivism to inform imputations might therefore improve assessments beyond what can be achieved in practice, hence we excluded it as a predictor of missing values.

Finally, to examine the preservation of STABLE-2007 and SARA-V2 scores across missing data conditions and techniques, we used descriptive statistics. This included means, standard deviations, and frequencies of both total scores.

#### Generating Missing Data Conditions

To examine the influence of missing data on predictive accuracy estimates, we used six missing data conditions. Condition 1 was the observed data, which were either virtually complete (STABLE-2007) or had low levels of missingness (SARA-V2). Conditions 2 to 6 generated missing data conditions, whereby 1% to 50% of MCAR data were inserted into the observed data in 10% increments (1–10, 11–20, 21–30, 31–40, 41–50). These missing data rates were chosen so that they (a) were distinct enough to identify potentially problematic levels of missingness, and (b) aligned with the standards for common sexual, domestic, and general violence risk tools. For instance, the VRS-SO allows 16% of items to be omitted, the SORAG allows 29% of items to be omitted, and the VRAG-R, DVRAG, and ODARA allow 33% to 38% of items to be omitted. See the Missing Data Generation section of the Online Supplement for procedural details, including procedures for one STABLE-2007 and three SARA-V2 items that were handled uniquely.

#### Predictive Accuracy

Harrell’s concordance index, or *c*-index, was used to examine the predictive accuracy of the STABLE-2007 and SARA-V2 (Harrell et al., 1982). It is the recommended effect size metric in risk assessment when the follow-up period is variable because it accounts for time at risk to reoffend (Hanson, 2022; Helmus & Babchishin, 2017). In the context of this study, the *c*-index can be interpreted as the probability that of two randomly selected individuals (at least one of whom reoffended), the one with the higher risk score reoffended first. The *c*-index can vary from 0 to 1, with .500 indicating no predictive discrimination (Harrell et al., 1996). Values of .556, .639, and .714 represent small, moderate, and large effect sizes, respectively (Helmus & Babchishin, 2017).

The *c*-index was obtained through Cox regression in R. The dependent variables for this analysis were time to sexual recidivism in years for STABLE-2007 total scores and time to domestic violence recidivism in years for SARA-V2 total scores. The proportional hazard assumption was tested using correlations between Schoenfeld residuals and time (Singer & Willett, 2003). All correlations were nonsignificant, indicating this assumption is met for both scales (STABLE-2007: *r*s = −.062 for mechanical totals and −.067 for prorated totals; SARA-V2: *r*s = .046 for mechanical totals and .034 for prorated totals). There were no univariate outliers on the SARA-V2, but there were 11 on the STABLE-2007 among nonrecidivists (*z*-scores = 3.30–3.73, exceeding the critical value of 3.29; Tabachnick & Fidell, 2014). These cases were retained, however, because *DFBETA* values for mechanical and prorated scores were below the size-adjusted cut-off of 0.031 (2/√ n; Belsley et al., 1980) and results for Condition 1 did not change after their removal. In addition, although unusually high, these were still in the range of possible scores.

Differences in predictive accuracy within and across missing data conditions were examined using confidence intervals and magnitude cut-offs of .556, .639, and .714. Namely, a missing data handling technique was considered meaningfully better than another if (a) it produced categorically better predictive accuracy, and (b) confidence intervals were not overlapping (*p* < .01; Cumming & Finch, 2005). Estimates from Condition 1 were used as the baseline for comparisons.

## Results

### Scale Descriptives

#### STABLE-2007

Scale descriptives for the STABLE-2007 are displayed in Table S1 of the Online Supplement. The overall missing data rate was 0.3%. Broken down by item, the percentage of missing data ranged from 0.0% to 0.5%. The overall distribution of item scores was positively skewed: 55.1% of item scores were 0 (no concern), 31.7% were 1 (some concern), and only 12.9% were 2 (considerable concern). Item means were similar, with one categorized as some concern (*M* = 1.2) and the rest categorized as no concern (*M*s = 0.2–0.8). Together, item distributions and means show that the sample generally presented with low to moderate sexual recidivism risk. They also appeared to respond similarly across items, indicating the equal item means assumption of proration is likely met.

Table 1 shows the missing data rates by case for the six missing data conditions. Recall that Condition 1 reflects the observed data, which were complete for 98.5% of the sample. Conditions 2 to 6 are the generated missing data conditions, whereby MCAR data were randomly inserted into the observed data in 10% increments (1–10, 11–20, 21–30, 31–40, 41–50). Across all conditions, 99% of the sample had the specified percentage of missing items, while the remaining 1% had more due to missingness in the observed data. Table S2 of the Online Supplement shows the percentage of missing data by item for each missing data condition.

**Table 1.** Percentage of Missing STABLE-2007 and SARA-V2 Items by Case Across Missing Data Conditions.

Risk scale	Condition 1 (observed data)	Condition 2 (1–10% missing)	Condition 3 (11–20% missing)	Condition 4 (21–30% missing)	Condition 5 (31–40% missing)	Condition 6 (41–50% missing)
% of cases | missing % of items						
STABLE-2007	98.5 | 0	98.6 | 8	98.7 | 15	48.7 | 23	98.9 | 39	99.0 | 46
	1.5 | 8–85	1.4 | 15–85	1.3 | 23–85	50.4 | 30.8	1.1 | 46–92	1.0 | 54–92
				0.9 | 39–85		
SARA-V2	82.4 | 0	45.7 | 5	46.6 | 15	47.0 | 25	47.9 | 35	48.8 | 45
	8.8 | 5	42.2 | 10	43.1 | 20	44.2 | 30	43.5 | 40	44.6 | 50
	4.0 | 10	6.4 | 15	5.7 | 25	4.2 | 35	4.6 | 45	3.3 | 55
	2.0 | 15	3.1 | 20	1.5 | 30	2.2 | 40	1.5 | 50	1.3 | 60
	2.9 | 20–55	2.6 | 25–60	3.1 | 35–65	2.4 | 45–70	2.4 | 55–75	2.0 | 65–80

*Note*. Condition 1 reflects the observed data. Conditions 2 to 6 reflect generated missing data conditions whereby 1%–50% of items per case were randomly deleted in 10% increments (1–10, 11–20, 21–30, 31–40, 41–50). Each cell reflects the percentage of cases that were missing the specified percentage of items for that condition. For instance, the top left cell indicates that 98.5% of cases were missing 0% of STABLE-2007 items in Condition 1 (98.5 | 0); the right adjacent cell indicates that 98.6% of cases were missing 8% of STABLE-2007 items in Condition 2 (98.6 | 8); and so on. Missing data rates exceed the target range for a small percentage of cases because the observed data were not complete (e.g., Condition 2 reflects 1%–10% missingness, but 1.4% of cases were missing 15%–85% of STABLE-2007 items, exceeding the 1%–10% target; this is because 1.5% of cases were missing 8%–85% of STABLE-2007 items in the observed data, or Condition 1). SARA-V2 = Spousal Assault Risk Assessment, Version 2.

#### SARA-V2

Scale descriptives for the SARA-V2 are displayed in Table S3 of the Online Supplement. The overall missing data rate was 2.1%. Broken down by item, the percentage of missing data ranged from 0.0% to 5.5%. The overall distribution of item scores was bimodal: 46.5% of scores were 0 (no/absent), 21.3% were 1 (possibly/partially present), and 30.1% were 2 (yes/present). Item means were fairly similar, with 12 categorized as no/absent (*M*s = 0.0–0.9) and eight categorized as possibly/partially present (*M*s = 1.0–1.5). Together, item distributions and means show that the sample generally presented with low to moderate domestic violence recidivism risk, but there were some higher-scoring individuals (particularly when compared to the STABLE-2007 sample). There is less support for the equal item means assumption of proration than seen with the STABLE-2007, but results do not appear unduly problematic. Table 1 shows the missing data rates by case for the six missing data conditions. Across generated missing data conditions, 88% to 93% of the sample had the specified percentage of missing items, while the remaining 7% to 12% had more due to missingness in the observed data. Table S4 of the Online Supplement shows the percentage of missing data by item for each missing data condition.

### Preservation of Risk Scores

The complete data in Condition 1 produced an average STABLE-2007 score of 7.5 (see Table 2). The average mechanical total score decreased by 3.7 points from Conditions 1 to 6 (three-quarters of a standard deviation), the average prorated total score decreased by 0.4 points, and average multiply imputed scores decreased by 0.01 to 0.02 points. A similar pattern was seen with the SARA-V2. The complete data in Condition 1 produced average SARA-V2 scores of 16.3 to 16.8 depending on the technique (see Table 2). The average mechanical total score decreased by 7.7 points from Conditions 1 to 6, the average prorated total score decreased by 0.1 points, and the average multiply imputed scores increased by 0.4 to 0.8 points. Thus, proration and multiple imputation preserve total scores as the missing data rate increases, whereas mechanical totals result in a marked reduction of absolute risk. The distributions of STABLE-2007 and SARA-V2 total scores for each missing data condition and technique are shown in Figures S1 and S2 of the Online Supplement, respectively.

**Table 2.** Preservation of STABLE-2007 and SARA-V2 Scores Across Missing Data Conditions and Techniques.

Condition	STABLE-2007 total score^a^ *M* (*SD*)				SARA-V2 total score^b^ *M* (*SD*)			
	Mechanical	Proration	MI_restrictive_	MI_inclusive_	Mechanical	Proration	MI_restrictive_	MI_inclusive_
1	7.47 (4.81)	7.50 (4.83)	7.50 (4.82)	7.50 (4.83)	16.31 (6.68)	16.73 (6.84)	16.76 (6.85)	16.75 (6.82)
2	6.87 (4.46)	7.47 (4.94)	7.50 (4.82)	7.50 (4.82)	15.17 (6.28)	16.77 (6.88)	16.80 (6.81)	16.80 (6.79)
3	6.27 (4.12)	7.45 (4.90)	7.50 (4.83)	7.51 (4.83)	13.47 (5.72)	16.71 (6.99)	16.79 (6.83)	16.83 (6.79)
4	5.37 (3.62)	7.38 (4.98)	7.53 (4.83)	7.53 (4.83)	11.95 (5.14)	16.78 (7.12)	16.96 (6.81)	17.06 (6.83)
5	4.46 (3.10)	7.35 (5.05)	7.52 (4.86)	7.53 (4.85)	10.25 (4.51)	16.73 (7.30)	17.05 (6.75)	17.25 (6.68)
6	3.82 (2.72)	7.15 (5.01)	7.48 (4.79)	7.49 (4.79)	8.57 (4.09)	16.59 (7.83)	17.12 (6.83)	17.56 (6.75)

*Note*. SARA-V2 = Spousal Assault Risk Assessment, Version 2; Condition 1 = observed data (mostly complete); Condition 2 = 1%–10% of items randomly deleted per case; Condition 3 = 11%–20% of items randomly deleted per case; Condition 4 = 21%–30% of items randomly deleted per case; Condition 5 = 31%–40% of items randomly deleted per case; Condition 6 = 41%–50% of items randomly deleted per case; Mechanical = mechanical total; Proration = prorated total; MI_restrictive_ = multiple imputation models with missing data correlates only (if applicable—STABLE-2007 was virtually complete, thus no missing data correlates were used); MI_inclusive_ = multiple imputation models with all auxiliary variables (missing data correlates if applicable and correlates of scale items).

a STABLE-2007 scores range from 0 to 26 for people with a child sex offense victim and 0 to 24 for everyone else. ^b^SARA-V2 total scores range from 0 to 40.

### Relative Predictive Accuracy

Relative predictive accuracy results for both scales are displayed in Table 3. The STABLE-2007 significantly predicted sexual recidivism across all models with moderate effects (*c*-indexes = .644–.672). Confidence intervals also overlapped across models, meaning that the three missing data handling techniques performed similarly (*p* > .01). The SARA-V2 significantly predicted domestic violence recidivism across all models with small effects (*c*-indexes = .582–.604), and confidence intervals overlapped, again indicating that mechanical totals, proration, and multiple imputation discriminated recidivists from nonrecidivists with comparable accuracy (*p* > .01).

**Table 3.** Predictive Accuracy of the STABLE-2007 and SARA-V2 Across Missing Data Conditions and Techniques.

Predictor	Condition 1	Condition 2	Condition 3	Condition 4	Condition 5	Condition 6
	*c*-index [95% CI]	*c*-index [95% CI]	*c*-index [95% CI]	*c*-index [95% CI]	*c*-index [95% CI]	*c*-index [95% CI]
STABLE-2007						
Mechanical	.670 [.633, .706]	.669 [.633, .705]	.666 [.629, .703]	.672 [.635, .709]	.652 [.613, .690]	.653 [.617, .689]
Proration	.669 [.633, .705]	.668 [.632, .704]	.665 [.628, .701]	.670 [.633, .707]	.652 [.614, .690]	.652 [.616, .687]
MI_restrictive_	.669 [.632, .704]	.669 [.632, .704]	.665 [.627, .701]	.659 [.619, .696]	.648 [.607, .687]	.644 [.604, .683]
MI_inclusive_	.669 [.632, .704]	.671 [.634, .706]	.670 [.632, .705]	.667 [.627, .704]	.663 [.623, .701]	.662 [.621, .700]
SARA-V2						
Mechanical	.592 [.544, .640]	.598 [.550, .647]	.601 [.552, .649]	.598 [.549, .646]	.594 [.546, .641]	.604 [.555, .652]
Proration	.586 [.537, .634]	.589 [.540, .638]	.591 [.541, .640]	.588 [.540, .637]	.587 [.539, .634]	.595 [.546, .644]
MI_restrictive_	.584 [.535, .632]	.586 [.536, .634]	.579 [.528, .628]	.582 [.530, .632]	.585 [.534, .635]	.582 [.528, .634]
MI_inclusive_	.584 [.535, .632]	.589 [.537, .636]	.584 [.534, .633]	.586 [.535, .636]	.585 [.535, .634]	.602 [.550, .653]

*Note.* All models are statistically significant (*p* < .05). SARA-V2 = Spousal Assault Risk Assessment, Version 2; Condition 1 = observed data (mostly complete); Condition 2 = 1%–10% of items randomly deleted per case; Condition 3 = 11%–20% of items randomly deleted per case; Condition 4 = 21%–30% of items randomly deleted per case; Condition 5 = 31%–40% of items randomly deleted per case; Condition 6 = 41%–50% of items randomly deleted per case; Mechanical = mechanical total; Proration = prorated total; MI_restrictive_ = multiple imputation models with missing data correlates only (if applicable—STABLE-2007 was virtually complete, thus no missing data correlates were used); MI_inclusive_ = multiple imputation models with all auxiliary variables (missing data correlates if applicable and correlates of scale items).

## Discussion

Missing data are pervasive in risk assessment but their impact on the predictive accuracy of risk tools has been largely unknown. A common technique for calculating risk scores when scale items are missing is to simply use the available items, either through summation (a mechanical total) or proration. Although frequently used in corrections, these methods have serious limitations. Multiple imputation is a more statistically defensible approach for dealing with missing item data that has not been tested against other techniques for predicting recidivism (apart from one study, which has various methodological limitations; Viljoen et al., 2017). In the current study, we compared the validity of these methods across different missing data conditions using two common sexual and domestic violence risk tools (STABLE-2007 and SARA-V2).

The results contradicted our hypotheses. As missing data increased, we expected predictive accuracy to decrease for mechanical and prorated scores but to remain stable for multiply imputed scores. We also expected multiple imputation to perform meaningfully better than mechanical totals and proration at moderate to high levels of missingness (11%–50% missingness), and proration to perform marginally better than mechanical totals. Instead, the STABLE-2007 significantly predicted sexual recidivism with a moderate effect within and across conditions, and confidence intervals overlapped across missing data handling techniques, showing comparable predictive accuracy. The SARA-V2 significantly predicted domestic violence recidivism with a small effect within and across conditions, and confidence intervals also overlapped across missing data handling techniques. On a statistical note, methodologists advocate for an inclusive imputation strategy that utilizes many auxiliary variables to predict missing values (e.g., Collins et al., 2001), but we found a restrictive strategy to perform similarly.

The good performance of mechanical totals was especially surprising because they produced the largest changes in risk scores across conditions. Namely, average STABLE-2007 and SARA-V2 mechanical scores decreased from Conditions 1 to 6 by 49% and 47%, respectively. Despite this drop in absolute risk score interpretations, there was no change in relative predictive accuracy. This illustrates that discrimination and calibration are two quite distinct properties of risk assessment scales (Hanson, 2022); discrimination accuracy can remain stable despite meaningful changes in calibration (Helmus et al., 2012). Consequently, although discrimination accuracy was robust across all methods for handling missing data, the results of the score preservation analyses suggest that mechanical totals would lead to a meaningful underestimation of absolute risk, particularly as the amount of missing information increases.

The unexpectedly robust findings for discrimination accuracy under diverse missing data conditions may suggest that the risk assessment scales examined are well-saturated (or possibly even over-saturated) with relevant risk factors. These scales do not purport to measure all relevant risk factors. However, it is expected that risk factors will be at least somewhat inter-correlated. Consequently, at some point, additional items (even of seemingly distinct risk factors) will reach a point of diminishing returns (Helmus, 2021). These findings may suggest that the STABLE-2007 and SARA-V2 have erred on the side of including more items than necessary, reaching some kind of plateau in predictive accuracy.

The use of mechanical and prorated totals should also be problematic for the SARA-V2 because tests of missing data correlates revealed that items in Condition 1 were, at best, MAR (see the Auxiliary Variables section of the Online Supplement). This should introduce bias into the results because these techniques assume data are MCAR (the assumption of equal item means for proration appears to be met). Despite these assumption violations, the predictive accuracy of the SARA-V2 was similar across missing data techniques. Finally, multiply imputed SARA-V2 Items 8–10 demonstrated distributional discrepancies (discussed in the Model Diagnostics section of the Online Supplement), which should impact the results; however, scale means and predictive accuracy for multiply imputed scores were comparable to the (mostly) complete data results in Condition 1, indicating no evidence of bias for multiple imputation.

### Limitations and Future Directions

Our unexpected findings are likely the result of two interrelated issues. First, we applied the missing data techniques across all individuals in our sample rather than just a subset. To the extent to which the missing data techniques biased total scores, the full sample received an equal amount of error. Future research should therefore examine the impact of these missing data techniques when applied to a subset of participants. This will better test if risk assessment results based on all scale items lead to more accurate predictions than those based on a subset of items. Second, we tested the models using a rank ordering statistic assessing discrimination (Harrell’s C), which would be less impacted than analyses of calibration. Thus, future research should also examine the impact of missing data and different missing data handling techniques on calibration.

The current sample is considered reflective of routine correctional populations (Hanson et al., 2016; Helmus et al., 2021), but the generalizability of findings for the SARA-V2 is limited to individuals with both sexual and domestic violence offending histories. Future research should therefore use a more representative domestic violence sample to enhance generalizability. Samples with a larger proportion of higher-risk cases are also needed to further validate the use of mechanical totals; our sample had relatively low STABLE-2007 and SARA-V2 item scores, meaning the drastic underestimation of risk though mechanical totals may not have been unduly problematic for these individuals. Findings could differ among higher-scoring individuals, especially for calibration statistics. Finally, the data we used had small amounts of missingness (1.0%–15.9% before data cleaning and 0.3%–2.1% after, discussed in the Missing Data Generation section of the Online Supplement), which introduces some noise to our results.

The missing data generation method we used also deleted items uniformly. Future research should systematically delete the strongest versus weakest predictors of recidivism to see if some items can tolerate more missing data than others. Starting with complete data and generating MAR and MNAR missing data mechanisms would also determine whether systematic missingness influences the predictive accuracy of risk tools (recall that we inserted MCAR data into scales that already had some missing data, albeit a small amount).

Although the use of multiple imputation in risk assessment is limited, it is becoming a more popular missing data handling technique. An additional recommendation for researchers using this technique concerns the role of recidivism in imputations. A search of PsycInfo and PsycArticles for documents with the terms “multiple imputation” and “risk assessment” identified eight studies that used multiple imputation to address missing risk assessment data. This included studies with missing risk scale data (Hildebrand et al., 2013; Kroner & Yessine, 2013; Viljoen et al., 2017; Whiting et al., 2023), and studies with missing risk factors not from risk scales (Baskin-Sommers et al., 2016; Martin et al., 2015; Matlasz et al., 2020; Taylor, 2015). Only two of these studies specified the role of recidivism in the imputation model (Hildebrand et al., 2013; Whiting et al., 2023), and it was included as a predictor of missing risk data. Whiting et al. did not provide a justification for this decision, but Hildebrand et al. cited Allison (2002). Allison’s recommendation to impute missing values using the outcome variable is based partly on diagnostic research (Landerman et al., 1997). In the absence of simulation studies on prognostic assessments, however, it is unclear whether this recommendation should be applied to risk assessment. Exploratory results from this sample suggest that using recidivism to predict missing risk scores might not be unduly problematic (see Table S5 of the Online Supplement), but to avoid improving risk assessments beyond what can be achieved in practice, researchers should err on the side of caution and exclude recidivism as a predictor of missing risk scores.

### Implications for Risk Tool Users, Developers, and Researchers

Generally, when implementing a risk tool for applied evaluations, it is important to follow the guidance from the scale developers, including how to handle missing data. Deviations from recommended practice should be clearly noted and would require sound justification. Concerns about the applicability of the research to the case at hand (e.g., due to missing data) should be noted external to the scale results, as part of acknowledging the strengths and limitations of the assessment method used. Fortunately, these findings suggest that at least two commonly used risk tools are fairly robust to missing data, suggesting that evaluators need not be overly concerned about the impact of occasional missing item data on the relative predictive accuracy of the scale. However, while simply omitting items from the total score may not meaningfully impact relative predictive accuracy, it may result in an underestimate of absolute risk levels.

For risk scale developers who have not provided guidance on handling missing data, the results of the current study suggest that proration is robust and defensible to use. We also believe proration is preferable to ignoring missing data through a mechanical total, as it is less likely to underestimate absolute risk. Multiple imputation is a more advanced method that can be used for research purposes (e.g., it requires information across a sample of individuals), but it cannot be used for an applied assessment of a single person. Despite the purported advantages of multiple imputation over simpler techniques, however, the current results suggest that prorated total scores are generally appropriate for handling missing data in research validation studies.

## Conclusions and Recommendations

Both the STABLE-2007 and SARA-V2 seem to tolerate missing data well and multiple imputation does not offer a clear advantage for discrimination accuracy over summing or prorating available items when applied across the full sample. Current practice for these scales is therefore defensible until more research is conducted, although users should be aware of how large amounts of missing data without proration or multiple imputation may substantially impact the interpretation of total scores. Deleting cases with missing data reduces statistical power and can introduce bias into the results if there is systematic missingness (Little & Rubin, 2002). Hence, it seems preferable to retain cases with missing data and, at a minimum, calculate prorated risk scores. Proration is simple and preserves total scores better than summing available items through a mechanical total. Until findings from the current study are replicated (i.e., showing that there is indeed no benefit of advanced techniques over simpler ones), researchers might also opt for multiple imputation given its stronger theoretical basis.

As a final note, the results presented here have stronger implications for research than for practice. Deleting 41% to 50% of items did not reduce relative predictive accuracy in the current study, but this does not mean decision-makers should apply STABLE-2007 or SARA-V2 results that are based on half the items. Suppose, for example, that a recidivist’s true score on the STABLE-2007 was 20, but due to missing data, their observed score was 10. Now suppose that a nonrecidivist’s true score was 10 and they had complete data. Our findings might seem to suggest that discrimination accuracy would not be affected if these scores were used to assess recidivism risk; however, case management decisions in the current study were based on mostly complete data. Instead, our results suggest that researchers need not delete cases with missing data, as is commonly done in risk assessment via listwise or pairwise deletion (e.g., Ferguson et al., 2009; Gray & Viljoen, 2023; Quinsey et al., 2006; Smid et al., 2014; Viljoen et al., 2017).

## Supplemental Material

sj-pdf-1-asm-10.1177_10731911231225191 – Supplemental material for The Effect of Missing Item Data on the Relative Predictive Accuracy of Correctional Risk Assessment Tools Supplemental material, sj-pdf-1-asm-10.1177_10731911231225191 for The Effect of Missing Item Data on the Relative Predictive Accuracy of Correctional Risk Assessment Tools by Bronwen Perley-Robertson, Kelly M. Babchishin and L. Maaike Helmus in Assessment

## Notes

1. The STABLE-2007 was virtually complete (0.3% missingness), thus no missing data correlates were needed.
