# Supplementary material for: The Effect of Missing Item Data on the Relative Predictive Accuracy of Correctional Risk Assessment Tools
Source: Assessment. 2024 Feb 7;31(8):1643–57. doi: 10.1177/10731911231225191 (PMC11490059; doi:10.1177/10731911231225191)
Supplement: sj-pdf-1-asm-10.1177_10731911231225191 – Supplemental material for The Effect of Missing Item Data on the Relative Predictive Accuracy of Correctional Risk Assessment Tools [file sj-pdf-1-asm-10.1177_10731911231225191.pdf]

The Effect of Missing Item Data on the Relative Predictive Accuracy of Correctional Risk Assessment Tools

Online Supplemental Material

Bronwen Perley-Robertson, Kelly M. Babchishin, and L. Maaike Helmus

Table of Contents

Multiple Imputation 3

Model Diagnostics 4

Auxiliary Variables 6

Missing Data Generation 8

References 10

Table S1: *Scale Descriptives for the STABLE-2007 in Condition 1 (Distribution of Item Scores, Item Means, and Missing Data Rates)* 11

Table S2: *Number and Percentage of Missing STABLE-2007 Items by Missing Data Condition* 12

Table S3: *Scale Descriptives for the SARA-V2 in Condition 1 (Distribution of Item Scores, Item Means, and Missing Data Rates)* 13

Table S4: *Number and Percentage of Missing SARA-V2 Items by Missing Data Condition* 14

Table S5: *Predictive Accuracy of the STABLE-2007 and SARA-V2 with Recidivism Used to Impute Missing Values* 15

Figure S1: *Mechanical and Prorated STABLE-2007 Scores Decrease as the Missing Data Rate Increases, with Mechanical Scores the Most Affected* 16

Figure S2: *Mechanical SARA-V2 Scores Decrease as the Missing Data Rate Increases* 17

Multiple Imputation

Multiple imputation was conducted at the item level using chained equations (i.e., fully conditional specification or sequential regression imputation; Raghunathan et al., 2001; van Buuren, 2007; van Buuren et al., 2006; van Buuren & Groothuis-Oudshoorn, 2011). This technique fills in missing data one variable at a time using an iterative process that matches the imputation model to each variable's distributional form. For instance, linear regression is used to impute continuous variables, logistic regression is used to impute binary variables, and proportional odds logistic regression is used to impute ordinal variables. Each regression model defines a distribution of plausible replacement values for the incomplete variable based on the predictors and a residual term. Replacement values are then drawn at random from this distribution. This imputation process is performed sequentially, starting with the most complete variable, and each sequence of imputations informs the next (i.e., after an incomplete variable is imputed, it is used as a predictor for every other incomplete variable in the chain). Once the entire dataset is filled in, a Bayesian procedure is used to sample a new set of regression parameters, which are used to generate the next set of imputations. Of note, each iteration uses the imputations from the previous cycle to predict missing values. This process continues until the distributions of the parameter estimates are stable across iterations (i.e., convergence), with the final iteration providing the data for analysis.

In the current study, chained equations was conducted in R using the *multivariate imputation by chained equations* (MICE) package (van Buuren & Groothuis-Oudshoorn, 2011). MICE employs parallel data augmentation, wherein *m* imputation chains that run for *t* iterations generate one imputed dataset each. This contrasts sequential data augmentation, wherein one long imputation chain saves *m* imputed datasets at every *t*th iteration (Enders, 2010). Following

recommendations by methodologists, we used a minimum of 20 imputations to meet power requirements (Graham et al., 2007) or matched the number of imputations to the percentage of cases with incomplete data when greater than 20% (Bodner, 2008; White et al., 2011). The number of iterations needed to reach convergence is typically much lower in MICE than other modern imputation techniques (e.g., joint modeling), which often require thousands of iterations (van Buuren & Groothuis-Oudshoorn, 2011).

Model Diagnostics

Model convergence was assessed using trace and potential scale reduction (PSR) factor plots. Trace plots in MICE display the mean and standard deviation for each imputed variable in the *m* chains plotted against the iteration number (van Buuren & Groothuis-Oudshoorn, 2011). Convergence is reached when there are no systematic changes in the parameters across iterations and the chains are intermingled. The PSR factor is a numeric diagnostic of the stability of parameter estimates across iterations (Gelman & Rubin, 1992). It essentially measures how much the imputations will improve with more iterations. Convergence is reached when PSR factor values are close to 1 (1.1 is the recommended threshold). Distributional discrepancy—the difference between observed and imputed data—was examined using density plots, which display the kernel density estimates (i.e., distribution) of the observed and imputed data (van Buuren, 2018). Distributions that align give a good indication that imputed data are plausible (van Buuren provides an example of a density plot for categorical variables; see Section 6.6.2).

We imputed 20 datasets in Condition 1 for both scales to meet power requirements, and 100 datasets in Conditions 2–6 to match the number of imputations to the percentage of incomplete cases. Additionally, we used 100 iterations for each imputation model. Trace plots for both scales showed no systematic changes in means or standard deviations across iterations

and chains were intermingled. For the SARA-V2, most PSR factor values were below 1.1 immediately or within a small number of iterations (5–10), whereas others dropped below this threshold by at least the 25th iteration. PSR factor values were slower to stabilize to 1 for conditions with more missing data, but this is to be expected (van Buuren & Groothuis-Oudshoorn, 2011). Together, the trace and PSR factor value plots provide a good indication of convergence for the SARA-V2.

Density plots generally showed good alignment between observed and imputed SARA-V2 values; however, imputed values for Items 8–10 were higher than observed values in Conditions 4–6 (21%–50% missingness), demonstrating distributional discrepancy. These results are not surprising, however, because missing values were filled in with 0s for roughly 75% of cases in the data cleaning stage (see the Missing Data Generation section below for more details). This resulted in 92%–98% of cases scoring a 0 on Items 8–10, compared to 15.4%–64.2% of cases scoring a 0 on the other items (see online supplementary Table S3). The multiple imputation procedure therefore skewed Items 8–10 upwards because it used observed values on the other items to predict missing values on Items 8–10. Nonetheless, the observed distributional discrepancies for these items did not appear to affect results (see results on the preservation of risk scores and predictive accuracy).

For the STABLE-2007, most PSR factor values were below 1.1 immediately, while others were below this threshold within a small number of iterations (5–10). PSR factor values were also quick to stabilize to 1, regardless of the missing data rate. Together, the trace and PSR factor plots provide a good indication of convergence for the STABLE-2007. Density plots showed good alignment between observed and imputed STABLE-2007 values, indicating no distributional discrepancies.

Auxiliary Variables

Methodologists recommend including correlates of missingness and the incomplete variables (i.e., auxiliary variables) in the imputation phase to (1) satisfy the MAR assumption, and (2) help recover some of the lost information (Baraldi & Enders, 2010; Collins et al., 2001; Enders, 2010). To identify correlates of missingness, we used a series of bivariate binary logistic regressions. These analyses treat missing data flags for each incomplete scale item as the dependent variable (1 = missing, 0 = complete) and possible correlates of missingness as the predictors (Boone et al., 2011). Variables that were significant at $p < .05$ were retained. Then, depending on their structure, we used different tests to identify correlates of the incomplete variables themselves (which are ordinal). For ordinal-ordinal and continuous-ordinal tests, we used Kendall's tau correlations. For binary-ordinal tests, we used a series of bivariate logistic regressions with STABLE-2007 and SARA-V2 items as the predictors. Variables significant at $p < .05$ for at least 20% of the items were included in the imputation phase for the relevant scale. We did not use an alpha correction for these analyses because we wanted a liberal test of auxiliary variables. The threshold of 20% was chosen to enhance imputations while also minimizing computational time.

Candidate auxiliary variables included demographics (age at the start of follow-up, Indigenous status, education level), severity of correctional sanctions (longest community supervision sentence ever received, both jail and community supervision as a sanction for the index offence), severity and extent of sexual and/or domestic violence offending history (number of prior domestic violence charges, number of prior sex offence charges, total number of sex offences ever recorded), Static-99R total score and items, and STABLE-2007 items for the

SARA-V2 sample (the reverse was not examined because only 10.6% of the STABLE-2007 sample had a SARA-V2 assessment).

Domestic violence offending history was only examined for the SARA-V2 sample because only 11.3% of the STABLE-2007 sample had a prior domestic violence offence. The number of prior sex offence charges was calculated from criminal history variables for the SARA-V2 sample and estimated from Item 5 of the Static-99R for the Stable-2007 sample (the required criminal history variables were not available in the latter sample). Static-99R total score and items were tested as correlates of missingness for SARA-V2 items, but only the total score was tested as a correlate of SARA-V2 items themselves, and only the total score and Item 5 (representing the number of prior sex offence charges) were tested as correlates of STABLE-2007 items. This ensured a rigorous test of missing data correlates to satisfy the MAR assumption for the SARA-V2, while also minimizing computational time needed for imputations. STABLE-2007 items were only tested as correlates of SARA-V2 items, not correlates of missingness, because there was no theoretical support for their utility beyond the variables already tested as correlates of missingness.

The following variables were identified as correlates of missingness for SARA-V2 items: longest community supervision sentence, Static-99R total score and Item 2 (*ever lived with a lover*), Item 4 (*prior nonsexual violence*), and Item 7 (*noncontact sex offence convictions*). This indicates that SARA-V2 items are at best MAR, violating the assumption of mechanical totals and proration. The following variables were significantly associated with at least 20% of SARA-V2 items: age, Indigenous, education, both jail and community supervision as a sanction, number of prior domestic violence charges, number of prior sex offence charges, and all STABLE-2007 items except Item 3.

As the STABLE-2007 was virtually complete, we only examined correlates of the items themselves. All candidate auxiliary variables were associated with at least 20% of STABLE-2007 items: age, Indigenous, education, longest community supervision sentence ever received, both jail and community supervision as a sanction for the index offence, total number of sex offences ever recorded, Static-99R total score, and Static-99R Item 5 (representing the number of prior sex offence charges).

Missing Data Generation

Missing data generation was performed separately by scale using the prodNA() function in the missForest package in R (Stekhoven & Bühlmann, 2012). Item 3 of the STABLE-2007 (*emotional identification with children*) was excluded from missing data generation because it is only scored for people with at least one child sex offence victim. STABLE-2007 total scores therefore range from 0 to 26 for people with a child victim and 0 to 24 for everyone else, which effectively means the latter group gets a 0 on Item 3 when calculating total scores. To preserve this nuance, 0s were imputed into missing Item 3 scores in the data cleaning stage, which reduced the overall missing data rate from 1.0% to 0.3%. This was also done to preserve the predictive accuracy of the STABLE-2007 given that Item 3 is a poor predictor of sexual recidivism for people with no child sex offence victims (Hanson et al., 2007). Allowing this item to be imputed could result in imputations above 0 for people with no child sex offence victims, which would inflate their total scores and potentially reduce predictive accuracy.

For the SARA-V2, Items 8–10 (*suicidal/homicidal ideation, psychotic/manic symptoms, personality disorder*) had 73.4% to 76.3% missingness, which is likely because they require professional mental health assessments to be scored and such assessments are rarely available in Canadian provincial corrections. Hence, individuals who were scored on these items likely had

the required assessments because there was enough cause for concern to request them. This suggests that Items 8–10 are missing not at random (MNAR; Rubin, 1976), with missing values reflecting an absence of risk, or a score of 0. Indeed, after imputing these items in MICE, density plots showed distributional discrepancies, which can be an indication of systematic missingness (van Buuren, 2018). We therefore filled in missing values on Items 8–10 with 0s in the data cleaning stage, which reduced the overall missing data rate from 15.9% to 2.1%.

References

* Additional references not in the manuscript.

Boone, L., Soenens, B., & Braet, C. (2011). Perfectionism, body dissatisfaction, and bulimic symptoms: The intervening role of perceived pressure to be thin and this idea internalization. *Journal of Social and Clinical Psychology*, *30*(10), 1043–1068. https://doi.org/10.1521/jscp.2011.30.10.1043

Gelman, A., & Rubin, D. B. (1992). Inference from iterative simulation using multiple sequences. *Statistical Science*, *7*(4), 457–472. https://doi.org/10.1214/ss/1177011136

Stekhoven, D. J., & Bühlmann, P. (2012). MissForest—non-parametric missing value imputation for mixed-type data. *Bioinformatics*, *28*(1), 112–118. https://doi.org/10.1093/bioinformatics/btr597

van Buuren, S. (2018). *Flexible imputation of missing data* (2nd ed.). Chapman and Hall/CRC. https://stefvanbuuren.name/fimd/

Table S1

Scale Descriptives for the STABLE-2007 in Condition 1 (Distribution of Item Scores, Item Means, and Missing Data Rates)

STABLE-2007 item	Item score 0: *n* (%)	1: *n* (%)	2: *n* (%)	*M* (SD)	Missing: *n* (%)
1. Significant social influences	2,225 (51.9)	1,443 (33.7)	599 (14.0)	0.6 (0.72)	19 (0.4)
2. Capacity for relationship stability	1,090 (25.4)	1,376 (32.1)	1,816 (42.4)	1.2 (0.81)	4 (0.1)
3. Emotional identification with children[a]	3,418 (79.7)	727 (17.0)	141 (3.3)	0.2 (0.50)	0 (0.0)
4. Hostility towards women	2,732 (63.7)	1,251 (29.2)	293 (6.8)	0.4 (0.62)	10 (0.2)
5. General social rejection/loneliness	1,847 (43.1)	1,931 (45.1)	497 (11.6)	0.7 (0.67)	11 (0.3)
6. Lack of concern for others	2,682 (62.6)	1,177 (27.5)	416 (9.7)	0.5 (0.67)	11 (0.3)
7. Impulsive acts	1,914 (44.7)	1,752 (40.9)	600 (14.0)	0.7 (0.70)	20 (0.5)
8. Poor cognitive problem-solving	1,545 (36.0)	2,051 (47.9)	671 (15.7)	0.8 (0.69)	19 (0.4)
9. Negative emotionality/hostility	2,881 (67.2)	1,024 (23.9)	363 (8.5)	0.4 (0.64)	18 (0.4)
10. Sex drive/sexual preoccupation	2,654 (61.9)	1,288 (30.1)	332 (7.7)	0.5 (0.64)	12 (0.3)
11. Sex as coping	2,921 (68.2)	1,018 (23.8)	332 (7.7)	0.4 (0.63)	15 (0.3)
12. Deviant sexual interests	1,884 (44.0)	1,633 (38.1)	757 (17.7)	0.7 (0.74)	12 (0.3)
13. Cooperation with supervision	2,905 (67.8)	1,014 (23.7)	354 (8.3)	0.4 (0.64)	13 (0.3)
Overall distribution of item scores[b]	30,698 (55.1)	17,685 (31.7)	7,171 (12.9)	-	164 (0.3)

Note. This table shows STABLE-2007 descriptives after data cleaning. Condition 1 = observed data (98.5% of cases complete); item score 0 = no concern; item score 1 = some concern; item score 2 = considerable concern; *N* = 4,286.

[a] This item is only scored for people with at least one child sex offence victim, meaning those with no child sex offence victims effectively get a 0 for the calculation of total scores. To avoid imputations greater than 0 for the latter group in Conditions 2–6, missing values were filled-in with 0s and this item was excluded from missing data generation.

[b] Of all possible item scores, 55.1% were 0, 31.7% were 1, 12.9% were 2, and 0.3% were missing.

Table S2

Number and Percentage of Missing STABLE-2007 Items by Missing Data Condition

	Missing data: *n* (%)					
STABLE-2007 item	Condition 1	Condition 2	Condition 3	Condition 4	Condition 5	Condition 6
1. Significant social influences	19 (0.4)	357 (8.3)	732 (17.1)	1,241 (29.0)	1,779 (41.5)	2,138 (49.9)
2. Capacity for relationship stability	4 (0.1)	377 (8.8)	707 (16.5)	1,282 (29.9)	1,812 (42.3)	2,188 (51.0)
3. Emotional identification with children[a]	0 (0.0)	0 (0.0)	0 (0.0)	0 (0.0)	0 (0.0)	0 (0.0)
4. Hostility towards women	10 (0.2)	353 (8.2)	722 (16.8)	1,255 (29.3)	1,756 (41.0)	2,145 (50.0)
5. General social rejection/loneliness	11 (0.3)	357 (8.3)	717 (16.7)	1,267 (29.6)	1,833 (42.8)	2,148 (50.1)
6. Lack of concern for others	11 (0.3)	362 (8.4)	713 (16.6)	1,230 (28.7)	1,778 (41.5)	2,116 (49.4)
7. Impulsive acts	20 (0.5)	396 (9.2)	731 (17.1)	1,293 (30.2)	1,825 (42.6)	2,167 (50.6)
8. Poor cognitive problem-solving	19 (0.4)	374 (8.7)	714 (16.7)	1,220 (28.5)	1,755 (40.9)	2,124 (49.6)
9. Negative emotionality/hostility	18 (0.4)	369 (8.6)	731 (17.1)	1,279 (29.8)	1,788 (41.7)	2,162 (50.4)
10. Sex drive/sexual preoccupation	12 (0.3)	368 (8.6)	721 (16.8)	1,241 (29.0)	1,794 (41.9)	2,140 (49.9)
11. Sex as coping	15 (0.3)	392 (9.1)	764 (17.8)	1,254 (29.3)	1,810 (42.2)	2,133 (49.8)
12. Deviant sexual interests	12 (0.3)	382 (8.9)	737 (17.2)	1,285 (30.0)	1,837 (42.9)	2,192 (51.1)
13. Cooperation with supervision	13 (0.3)	350 (8.2)	716 (16.7)	1,301 (30.4)	1,761 (41.1)	2,145 (50.0)

Note. This table shows STABLE-2007 descriptives after data cleaning. Condition 1 = observed data (98.5% of cases complete); Condition 2 = 1%–10% of items randomly deleted per case; Condition 3 = 11%–20% of items randomly deleted per case; Condition 4 = 21%–30% of items randomly deleted per case; Condition 5 = 31%–40% of items randomly deleted per case; Condition 6 = 41%–50% of items randomly deleted per case; *N* = 4,286.

[a] This item is only scored for people with at least one child sex offence victim, meaning those with no child sex offence victims effectively get a 0 for the calculation of total scores. To avoid imputations greater than 0 for the latter group in Conditions 2–6, missing values were filled-in with 0s and this item was excluded from missing data generation.

Table S3

Scale Descriptives for the SARA-V2 in Condition 1 (Distribution of Item Scores, Item Means, and Missing Data Rates)

	Item score				
SARA-V2 item	0: *n* (%)	1: *n* (%)	2: *n* (%)	*M* (SD)	Missing: *n* (%)
1. Past assault of family members	292 (64.2)	21 (4.6)	132 (29.0)	0.6 (0.91)	10 (2.2)
2. Past assault of strangers or acquaintances	195 (42.9)	27 (5.9)	223 (49.0)	1.1 (0.97)	10 (2.2)
3. Past violation of conditional release/supervision	168 (36.9)	15 (3.3)	268 (58.9)	1.2 (0.96)	4 (0.9)
4. Recent relationship problems	81 (17.8)	100 (22.0)	272 (59.8)	1.4 (0.78)	2 (0.4)
5. Recent employment problems	131 (28.8)	179 (39.3)	140 (30.8)	1.0 (0.78)	5 (1.1)
6. Victim of and/or witness to family violence	171 (37.6)	118 (25.9)	141 (31.0)	0.9 (0.85)	25 (5.5)
7. Recent substance abuse/dependence	105 (23.1)	189 (41.5)	155 (34.1)	1.1 (0.75)	6 (1.3)
8. Recent suicidal or homicidal ideation/intent	428 (94.1)	19 (4.2)	8 (1.8)	0.1 (0.33)	0 (0.0)
9. Recent psychotic and/or manic symptoms	444 (97.6)	7 (1.5)	4 (0.9)	0.0 (0.22)	0 (0.0)
10. Personality disorder (anger, impulsivity, instability)	418 (91.9)	10 (2.2)	27 (5.9)	0.1 (0.49)	0 (0.0)
11. Past physical assault	112 (24.6)	21 (4.6)	312 (68.6)	1.5 (0.87)	10 (2.2)
12. Past sexual assault/sexual jealousy	186 (40.9)	99 (21.8)	151 (33.2)	0.9 (0.88)	19 (4.2)
13. Past use of weapons and/or credible threats of death	229 (50.3)	86 (18.9)	129 (28.4)	0.8 (0.87)	11 (2.4)
14. Recent escalation in frequency or severity of assault	162 (35.6)	171 (37.6)	113 (24.8)	0.9 (0.78)	9 (2.0)
15. Past violation of “no contact” orders	278 (61.1)	41 (9.0)	117 (25.7)	0.6 (0.88)	19 (4.2)
16. Extreme minimization/denial of spousal assault	92 (20.2)	248 (54.5)	107 (23.5)	1.0 (0.67)	8 (1.8)
17. Attitudes that support or condone spousal assault	195 (42.9)	222 (48.8)	26 (5.7)	0.6 (0.60)	12 (2.6)
18. Severe and/or sexual assault	70 (15.4)	206 (45.3)	169 (37.1)	1.2 (0.70)	10 (2.2)
19. Use of weapons and/or credible threats of death	218 (47.9)	110 (24.2)	114 (25.1)	0.8 (0.84)	13 (2.9)
20. Violation of “no contact” order	256 (56.3)	50 (11.0)	134 (29.5)	0.7 (0.90)	15 (3.3)
Overall distribution of item scores[a]	4,231 (46.5)	1,939 (21.3)	2,742 (30.1)	-	188 (2.1)

Note. This table shows SARA-V2 descriptives after data cleaning. SARA-V2 = Spousal Assault Risk Assessment, Version 2; Condition 1 = observed data (82.4% of cases complete); item score 0 = no/absent; item score 1 = possibly/partially present; item score 2 = yes/present; *N* = 455.

[a] Of all possible item scores, 46.5% were 0, 21.3% were 1, 30.1% were 2, and 2.1% were missing.

Table S4

Number and Percentage of Missing SARA-V2 Items by Missing Data Condition

SARA-V2 item	Missing data: *n* (%)					
	Condition 1	Condition 2	Condition 3	Condition 4	Condition 5	Condition 6
1. Past assault of family members	10 (2.2)	42 (9.2)	80 (17.6)	120 (26.4)	170 (37.4)	207 (45.5)
2. Past assault of strangers or acquaintances	10 (2.2)	42 (9.2)	86 (18.9)	126 (27.7)	171 (37.6)	206 (45.3)
3. Past violation of conditional release/supervision	4 (0.9)	19 (4.2)	70 (15.4)	109 (24.0)	159 (34.9)	191 (42.0)
4. Recent relationship problems	2 (0.4)	37 (8.1)	90 (19.8)	133 (29.2)	174 (38.2)	227 (49.9)
5. Recent employment problems	5 (1.1)	51 (11.2)	90 (19.8)	134 (29.5)	165 (36.3)	228 (50.1)
6. Victim of and/or witness to family violence	25 (5.5)	59 (13.0)	95 (20.9)	137 (30.1)	190 (41.8)	241 (53.3)
7. Recent substance abuse/dependence	6 (1.3)	46 (10.1)	87 (19.1)	138 (30.3)	174 (38.2)	221 (48.6)
8. Recent suicidal or homicidal ideation/intent	0 (0.0)	39 (8.6)	87 (19.1)	131 (28.8)	163 (35.8)	209 (45.9)
9. Recent psychotic and/or manic symptoms	0 (0.0)	38 (8.4)	82 (18.0)	125 (27.5)	173 (38.0)	216 (47.5)
10. Personality disorder (anger, impulsivity, instability)	0 (0.0)	36 (7.9)	77 (16.9)	123 (27.0)	164 (36.0)	211 (46.4)
11. Past physical assault	10 (2.2)	41 (9.0)	94 (20.7)	136 (29.9)	190 (41.8)	241 (53.0)
12. Past sexual assault/sexual jealousy	19 (4.2)	48 (10.5)	96 (21.2)	139 (30.5)	184 (40.4)	220 (48.4)
13. Past use of weapons and/or credible threats of death	11 (2.4)	40 (8.8)	88 (19.3)	148 (32.5)	183 (40.2)	214 (47.0)
14. Recent escalation in frequency or severity of assault	9 (2.0)	40 (8.8)	87 (19.1)	124 (27.3)	166 (36.5)	215 (47.3)
15. Past violation of "no contact" orders	19 (4.2)	46 (10.1)	82 (18.0)	125 (27.5)	175 (38.5)	223 (49.0)
16. Extreme minimization/denial of spousal assault	8 (1.8)	44 (9.7)	99 (21.8)	135 (29.7)	181 (39.8)	221 (48.6)
17. Attitudes that support or condone spousal assault	12 (2.6)	38 (8.4)	82 (18.0)	130 (28.6)	167 (36.7)	215 (47.3)
18. Severe and/or sexual assault	10 (2.2)	46 (10.1)	84 (18.5)	128 (28.1)	185 (40.7)	218 (47.9)
19. Use of weapons and/or credible threats of death	13 (2.9)	38 (8.4)	70 (15.4)	141 (31.0)	190 (41.8)	241 (53.0)
20. Violation of "no contact" order	15 (3.3)	40 (8.8)	91 (20.0)	126 (27.7)	182 (40.0)	226 (49.7)

Note. This table shows SARA-V2 descriptives after data cleaning. SARA-V2 = Spousal Assault Risk Assessment, Version 2; Condition 1 = observed data (82.4% of cases complete); Condition 2 = 1%–10% of items randomly deleted per case; Condition 3 = 11%–20% of items randomly deleted per case; Condition 4 = 21%–30% of items randomly deleted per case; Condition 5 = 31%–40% of items randomly deleted per case; Condition 6 = 41%–50% of items randomly deleted per case; *N* = 455.

Table S5

Predictive Accuracy of the STABLE-2007 and SARA-V2 with Recidivism Used to Impute Missing Values

Predictor	Condition 1	Condition 2	Condition 3
	c-index [95% CI]	*c*-index [95% CI]	*c*-index [95% CI]
STABLE-2007[a]			
Mechanical	.62 [.515, .718]	.63 [.531, .731]	.63 [.524, .731]
Proration	.62 [.513, .717]	.63 [.526, .729]	.62 [.521, .722]
$MI_{restrictive}$	-	.64 [.537, .736]	.63 [.521, .731]
$MI_{inclusive}$	-	.65 [.543, .737]	.63 [.510, .727]
SARA-V2[b]			
Mechanical	.59 [.544, .640]	.60 [.548, .645]	.59 [.542, .639]
Proration	.59 [.539, .637]	.59 [.544, .642]	.59 [.544, .643]
$MI_{restrictive}$	.59 [.544, .641]	.58 [.528, .634]	.59 [.536, .647]
$MI_{inclusive}$	.59 [.540, .641]	.58 [.529, .634]	.59 [.538, .648]

Note. These results are from an earlier version of this manuscript that used (1) a smaller STABLE-2007 sample, (2) fewer and less distinct missing data conditions, and (3) recidivism to impute missing risk scale items. All models were statistically significant. SARA-V2 = Spousal Assault Risk Assessment, Version 2; Condition 1 = observed data (STABLE-2007 virtually complete; generally 0%–15% of SARA-V2 items missing per case); Condition 2 = 15.4%–23.1% and 20%–35% of STABLE-2007 and SARA-V2 items randomly deleted per case, respectively; Condition 3 = 30.8%–46.2% and 35%–50% of STABLE-2007 and SARA-V2 items randomly deleted per case, respectively; Mechanical = mechanical total; $MI_{restrictive}$ = multiple imputation models with missing data correlates and/or outcomes only (missing data correlates only used for the SARA-V2 as the STABLE-2007 was virtually complete); $MI_{inclusive}$ = multiple imputation models with all auxiliary variables.

[a] The STABLE-2007 (N = 467) was virtually complete, thus multiple imputation was not used for Condition 1 at this stage in the project.

[b] SARA-V2: N = 455.

Figure S1

Mechanical and Prorated STABLE-2007 Scores Decrease as the Missing Data Rate Increases, with Mechanical Scores the Most Affected

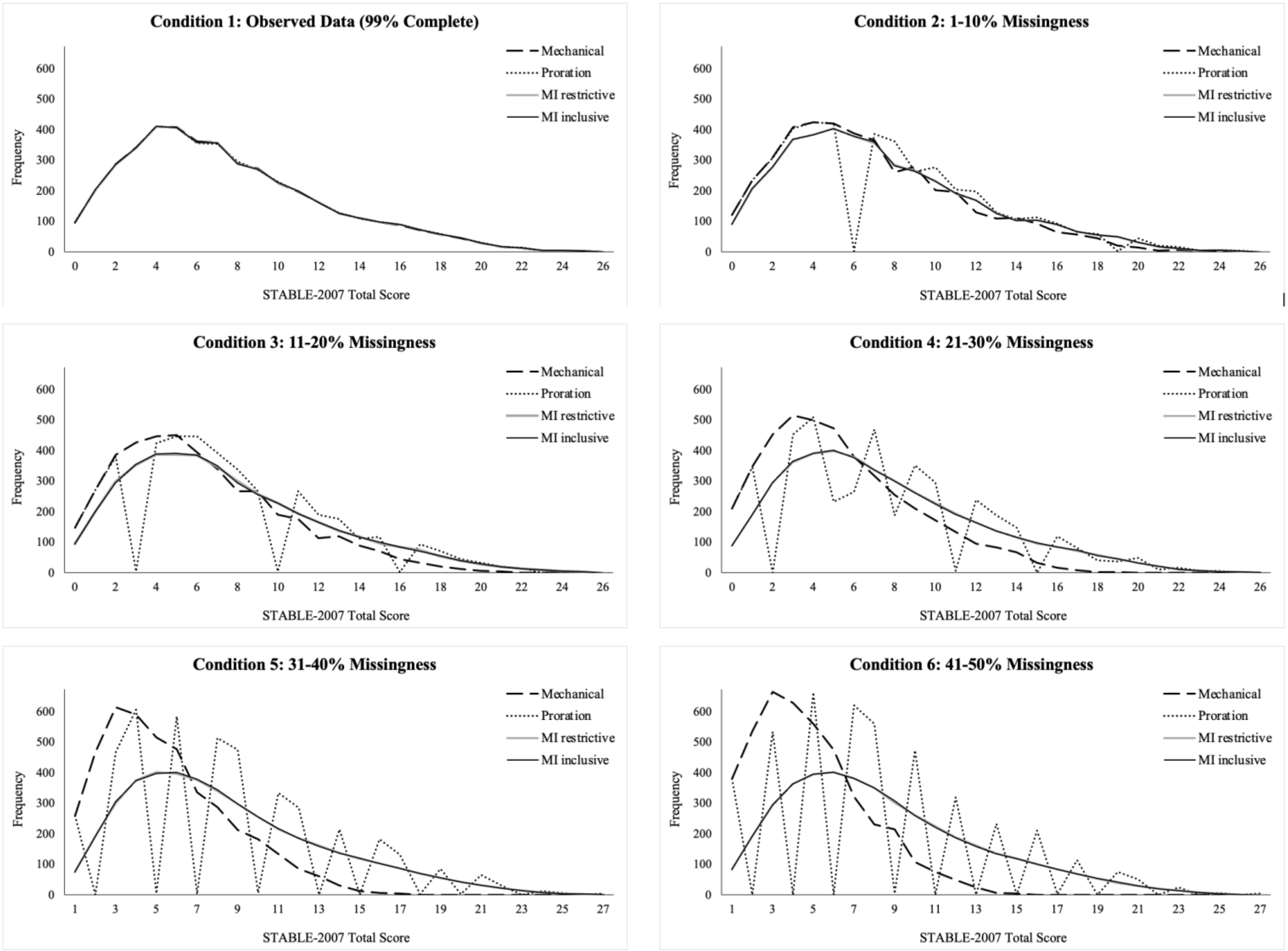

Note. The complete data in Condition 1 produced a positively skewed distribution. As the missing data rate increased, this positive skew became more pronounced for mechanical and prorated totals, with the former particularly affected. The distribution of multiply imputed total scores, however, remained remarkably stable across missing data conditions. Condition 1 = observed data (mostly complete); Condition 2 = 1%–10% of items randomly deleted per case; Condition 3 = 11%–20% of items randomly deleted per case; Condition 4 = 21%–30% of items randomly deleted per case; Condition 5 = 31%–40% of items randomly deleted per case; Condition 6 = 41%–50% of items randomly deleted per case. Mechanical = mechanical total; Proration = prorated total; MI restrictive = multiple imputation model with no auxiliary variables; MI inclusive = multiple imputation model with correlates of STABLE-2007 items; N = 4,286.

Figure S2

Mechanical SARA-V2 Scores Decrease as the Missing Data Rate Increases

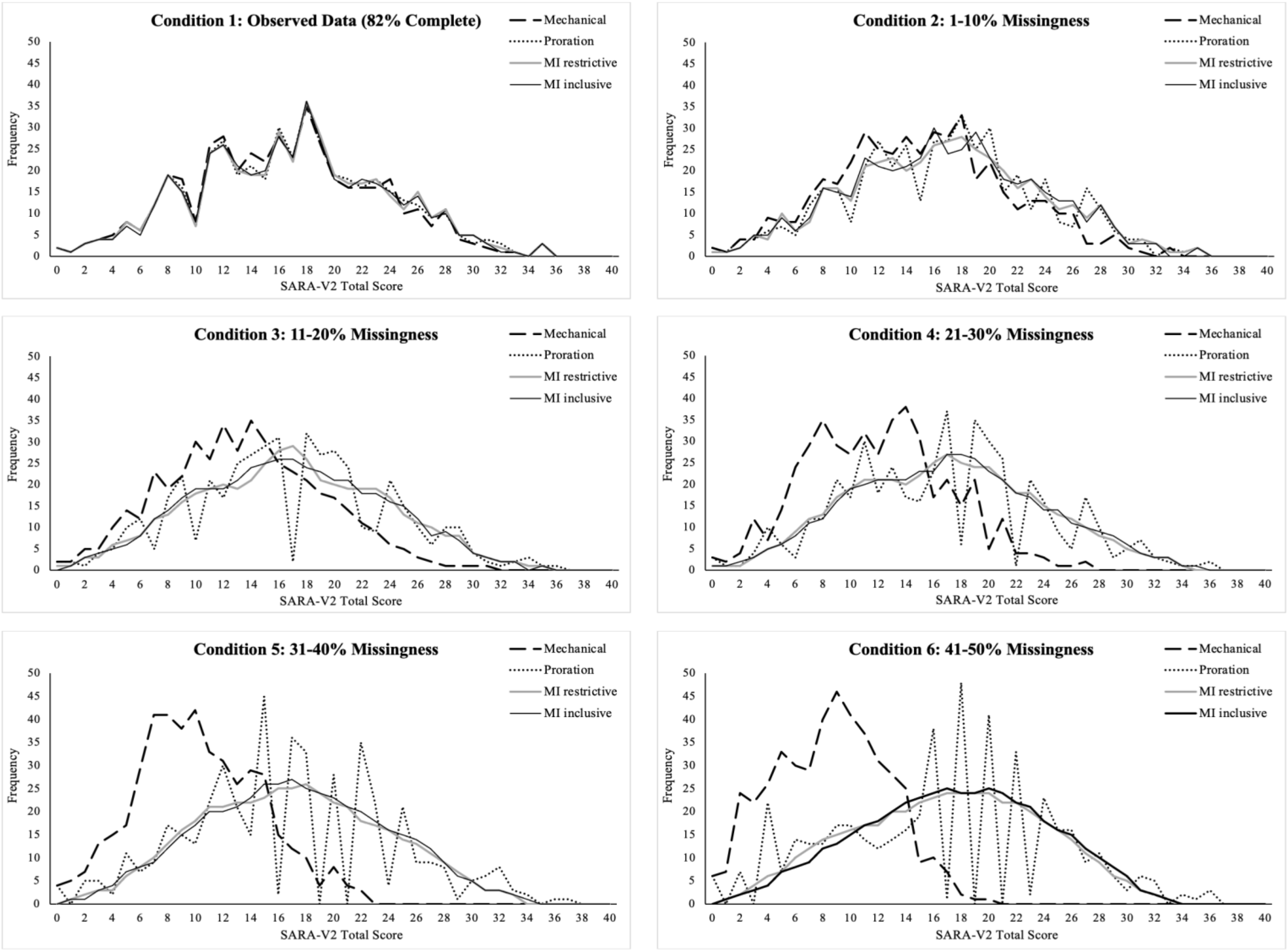

Note. All techniques produced similar results in Condition 1, with a slightly positively skewed distribution. This skew became more pronounced for mechanical totals as the missing data rate increased, while scores for the other three techniques became more normally distributed. SARA-V2 = Spousal Assault Risk Assessment, Version 2; Condition 1 = observed data (mostly complete); Condition 2 = 1%–10% of items randomly deleted per case; Condition 3 = 11%–20% of items randomly deleted per case; Condition 4 = 21%–30% of items randomly deleted per case; Condition 5 = 31%–40% of items randomly deleted per case; Condition 6 = 41%–50% of items randomly deleted per case. Mechanical = mechanical total; Proration = prorated total; MI restrictive = multiple imputation model with missing data correlates; MI inclusive = multiple imputation model with added correlates of SARA%–V2 items; *N* = 455.
